# Whole Genome Identification of Potential G-Quadruplexes and Analysis of the G-Quadruplex Binding Domain for SARS-CoV-2

**DOI:** 10.3389/fgene.2020.587829

**Published:** 2020-11-27

**Authors:** Rongxin Zhang, Ke Xiao, Yu Gu, Hongde Liu, Xiao Sun

**Affiliations:** State Key Laboratory of Bioelectronics, School of Biological Science and Medical Engineering, Southeast University, Nanjing, China

**Keywords:** G-quadruplex, SARS-CoV-2, COVID-19, G4, coronavirus, G-quadruplex binding domain, SUD-homology structure

## Abstract

The coronavirus disease 2019 (COVID-19) pandemic caused by SARS-CoV-2 (severe acute respiratory syndrome coronavirus 2) has become a global public health emergency. G-quadruplex, one of the non-canonical secondary structures, has shown potential antiviral values. However, little is known about the G-quadruplexes of the emerging SARS-CoV-2. Herein, we characterized the potential G-quadruplexes in both positive and negative-sense viral strands. The identified potential G-quadruplexes exhibited similar features to the G-quadruplexes detected in the human transcriptome. Within some bat- and pangolin-related betacoronaviruses, the G-tracts rather than the loops were under heightened selective constraints. We also found that the amino acid sequence similar to SUD (SARS-unique domain) was retained in SARS-CoV-2 but depleted in some other coronaviruses that can infect humans. Further analysis revealed that the amino acid residues related to the binding affinity of G-quadruplexes were conserved among 16,466 SARS-CoV-2 samples. Moreover, the dimer of the SUD-homology structure in SARS-CoV-2 displayed similar electrostatic potential patterns to the SUD dimer from SARS. Considering the potential value of G-quadruplexes to serve as targets in antiviral strategy, our fundamental research could provide new insights for the SARS-CoV-2 drug discovery.

## Introduction

The coronavirus disease 2019 (COVID-19) pandemic, which first broke out in China, has rapidly become a global public health emergency within a few months (Lai et al., 2020). According to the statistics from the Johns Hopkins Coronavirus Resource Center (https://coronavirus.jhu.edu/map.html), as of July 22, 2020, 15 million cases have been confirmed, with a death toll rising to 600,000. Since 2000, humans have suffered at least three coronavirus outbreaks, and they were severe acute respiratory syndrome (SARS) in 2003 (Zhong et al., 2003; Peiris et al., 2004; Zumla et al., 2016; Cui et al., 2019), Middle East respiratory syndrome (MERS) in 2012 (Zumla et al., 2016; Cui et al., 2019), and COVID-19. Scientists identified and sequenced the virus early in this outbreak, and named it SARS-CoV-2 (Gorbalenya et al., 2020). The symptoms of the patients infected with the novel coronavirus vary from person to person, and fever, cough, and fatigue are the most common ones (Guan et al., 2020; Jin et al., 2020; Rothan and Byrareddy, 2020; Zu et al., 2020). The clinical chest CT (computed tomography) and nucleic acid testing are the most typical methods of diagnosing COVID-19 (Jin et al., 2020; Zu et al., 2020). It is worth noting that the recent achievements in AI (artificial intelligence) aid diagnosis technology (Li et al., 2020) and CRISPR-Cas12-based detection methods (Broughton et al., 2020) are expected to expand the diagnosis of COVID-19. Despite the great efforts of the researchers, no specific clinical drugs or vaccines had developed to cope with COVID-19 by the end of September 2020.

**Figure 1:**
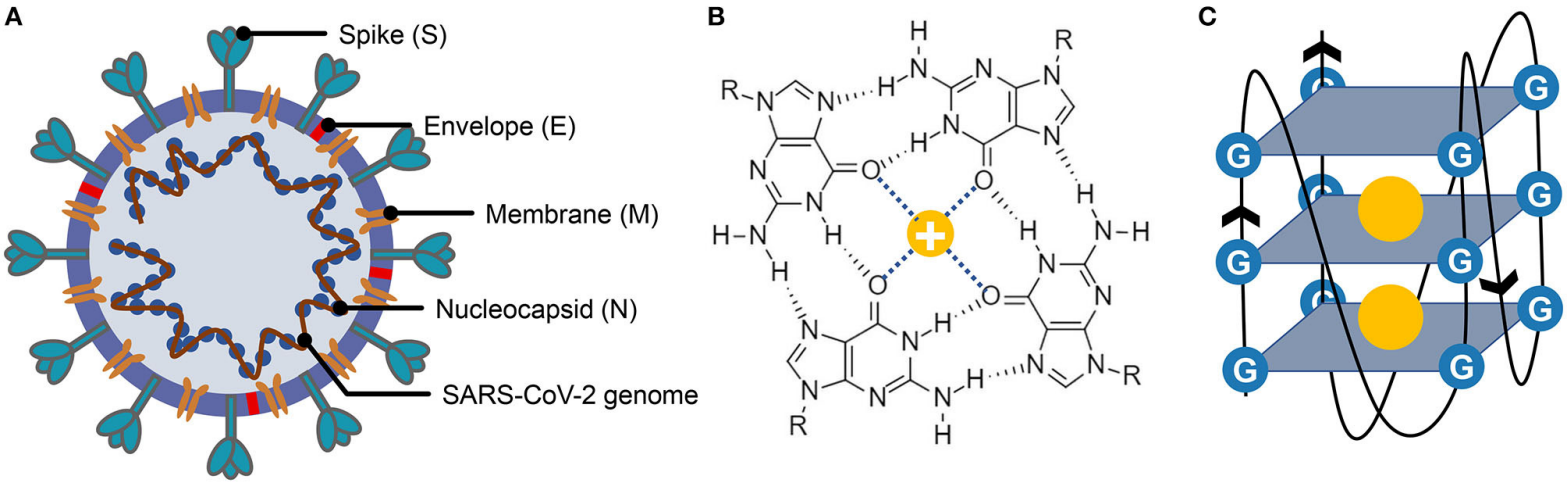
Structure of severe acute respiratory syndrome coronavirus 2 (SARS-CoV-2), G-quartet, and G-quadruplex. **(A)** The SARS-CoV-2 particle structure is composed of four structural proteins, which are the spike protein, the envelope protein, the membrane protein, and the nucleocapsid protein. The nucleocapsid proteins are bound to the SARS-CoV-2 genome. **(B)** Structure of G-quartet; the neighboring guanines are connected via Hoogsteen hydrogen. The cation is indicated by a yellow circle. **(C)** The G-quadruplex is formed by stacking multiple G-quartets, and the stabilization of the structure is partially determined by the central cations.

SARS-CoV-2 is a betacoronavirus within the Coronaviridae family that is the culprit responsible for the COVID-19 pandemic (Figure 1A) (Guo et al., 2020; Zheng, 2020). Studies have confirmed that SARS-CoV-2 is a positive-sense single-stranded RNA [(+)ssRNA] virus with a total length of approximately 30k. The positive-sense RNA strand of SARS-CoV-2 can serve as a template to produce viral proteins related to replication, structure composition, and other functions or events (Chen et al., 2020; Kim et al., 2020). One of the hotspots is how SARS-CoV-2 entry the host cells. SARS-CoV-2 has shown a great affinity to the angiotensin-converting enzyme 2 (ACE2), which has been proved to be the binding receptor for SARS-CoV-2 (Hoffmann et al., 2020; Walls et al., 2020). After entering the host cells, the viral genomic RNA will be released to the cytoplasm, and the ORF1a/ORF1ab is subsequently translated into replicase polyproteins of pp1a/pp1ab, which will be cleaved into some non-structural proteins (nsps). These non-structure proteins ultimately form the replicase–transcriptase complex for replication and transcription. Along with the full-length positive- and negative-sense RNAs, a nested set of subgenomic RNAs (sgRNAs) are also synthesized, and mainly translated into some structural proteins and accessory proteins. When the assembly is finished, the mature SARS-CoV-2 particles are released from the infected host cells via exocytosis (Shereen et al., 2020). Mounting evidence suggests that bats and pangolins are the suspected natural host and intermediate host of SARS-CoV-2 (Andersen et al., 2020; Lam et al., 2020; Zhang T. et al., 2020; Zhou et al., 2020). Intriguingly, a report from Yongyi Shen et al. showed that SARS-CoV-2 might be the recombination product of Bat-CoV-RaTG13-like virus and Pangolin-CoV-like virus (Xiao et al., 2020).

G-quadruplexes are the non-canonical nucleic acid structures usually formed in G-rich regions both in DNA and RNA strands (Bochman et al., 2012; Kwok and Merrick, 2017; Varshney et al., 2020). The G-quadruplex is formed by stacking G-quartets (Figure 1B) on top of each other, in which the four guanines making up a G-quartet are connected via Hoogsteen pairs (Figure 1C) (Bochman et al., 2012; Kwok and Merrick, 2017; Spiegel et al., 2020; Varshney et al., 2020). Extensive research indicated that G-quadruplexes were involved in many critical biological processes, including DNA replication (Hoshina et al., 2013; Valton et al., 2014; Valton and Prioleau, 2016; Prorok et al., 2019), telomere regulation (Tang et al., 2007; Wang et al., 2011; Takahama et al., 2013; Moye et al., 2015; Jansson et al., 2019), and RNA translation (Kumari et al., 2007; Gomez et al., 2010; Murat et al., 2018; Jodoin et al., 2019). It has been proven that G-quadruplexes existed in the viral genome and can regulate the viral biological processes, which made it possible to function as potential drug targets for antiviral strategy (Métifiot et al., 2014; Ruggiero and Richter, 2018; Saranathan and Vivekanandan, 2019). A study made by Jinzhi Tan et al. demonstrated that the SARS-unique domain within the nsp3 (nonstructural protein 3) of SARS coronavirus (SARS-CoV) exhibits the binding preference to the G-quadruplex structure in the human transcript and potentially interfere with host cell antiviral response (Tan et al., 2009). They also identified several amino acid residues that were tightly associated with its binding capacity. Yet, whether the SARS-CoV-2 contains a G-quadruplex binding domain and whether the amino acid residues related to the G-quadruplex binding affinity are conserved among SARS-CoV-2 samples need further interpretation. Besides, the G-quadruplexes in some well-known virus, such as HIV-1 (human immunodeficiency viruses type 1) (Perrone et al., 2014; Piekna-Przybylska et al., 2014; Butovskaya et al., 2018, 2019), ZIKV (ZIKA virus) (Fleming et al., 2016), HPV (human papillomavirus) (Tlučková et al., 2013; Marušič et al., 2017) and EBOV (Ebola virus) (Wang et al., 2016a) have been studied. However, in our understanding of the G-quadruplexes, their potential roles in the emerging SARS-CoV-2 are lacking.

In this study, we depicted the potential G-quadruplexes (PG4s) in SARS-CoV-2 by merging several G-quadruplex prediction tools. The PG4s in SARS-CoV-2 presented similar features to the two-quartet G-quadruplexes in the human transcriptome, which potentially supported the formation and existence of the G-quadruplexes in SARS-CoV-2. Additionally, we investigated the difference in selective constraints between the G-tracts and other nucleotides in the SARS-CoV-2 genome. To further elucidate the possible pathogenic mechanism of SARS-CoV-2, we examined the sequence and structure of the SUD-homology in SARS-CoV-2 that are critical in binding the G-quadruplex structures in host transcripts.

## Materials and Methods

### Data Collection

We obtained a total of 77 full-length bat-associated betacoronaviruses from the DBatVir (http://www.mgc.ac.cn/DBatVir/) database (Supplementary Table 1) (Chen et al., 2014). We also downloaded the bat coronavirus RaTG13 (MN996532.1) genome from the NCBI virus database (https://www.ncbi.nlm.nih.gov/labs/virus/vssi/#/), which has shown a high sequence similarity to the SARS-CoV-2 reference genome in previous reports. We acquired the SARS-CoV-2 reference genome from the NCBI virus database under the accession number of NC_045512. In addition to those sequences, nine pangolin coronaviruses were derived from GISAID (https://www.gisaid.org/) database (Shu and McCauley, 2017). To calculate the mutation frequency of each nucleotide in SARS-CoV-2, we downloaded the SARS-CoV-2 alignment sequence file from the GISAID database spanning from December 24, 2019, to April 24, 2020, which contains 16,466 SARS-CoV-2 samples. The ORF1ab amino acid sequences of 24 viruses were retrieved from the NCBI Protein database (https://www.ncbi.nlm.nih.gov/protein/). The detailed accession numbers of the sequence data used in this study are described in Supplementary Tables 1, 2.

### Pairwise and Multiple Sequence Alignment, Phylogenetic, and Conservation Analysis

The EMBOSS Needle software, which is based on the Needleman–Wunsch algorithm and is a part of the EMBL-EBI web tools (Madeira et al., 2019), was employed for the pairwise sequence alignment. Clustal Omega (Sievers et al., 2011; Sievers and Higgins, 2018) is a reliable and accurate multiple sequence alignment (MSA) tool that can be performed on large data sets. We utilized this MSA tool to align the viral genomes and the protein sequences under the default paraments, respectively. UGENE (Okonechnikov et al., 2012) is a powerful and user-friendly bioinformatics software, and we choose UGENE to visualize the pairwise and multiple sequence alignment results. We used the MEGA X software (Kumar et al., 2018) to construct the neighbor-joining phylogenetic tree with 1,000 bootstrap replications. To depict the conservation state for each nucleotide site, the GERP++ software (Davydov et al., 2010) was applied to calculate the “Rejected Substitutions” score column by column, which can reflect the strength of constraints for each nucleotide site. The key software or databases are listed in the Supplementary Table 3.

### Potential G-Quadruplex Detection

Several open-source G-quadruplex detection software were used to search the PG4s both in the SARS-CoV-2 positive- and negative-sense strands. G4CatchAll (Doluca, 2019), pqsfinder (Hon et al., 2017), and QGRS Mapper (Kikin et al., 2006) were employed to predict the putative G-quadruplexes, respectively; please see ref (Puig Lombardi and Londoño-Vallejo, 2019) for more information about the comparison of those tools mentioned above. The minimum G-tract length was set to two in the three pieces of software, while the max length of the predicted G-quadruplexes was limited to 30. Specifically, the minimum score of the predicted G-quadruplex was set to 10 when using pqsfinder. We utilized BEDTools (Quinlan and Hall, 2010) to sort the PG4s according to their coordinates. Apart from this, we adopted the cG/cC scoring system (Beaudoin et al., 2014) proposed by Jean-Pierre Perreault et al. to delineate the influence of sequence context on PG4s. The PG4s along with 15-nt (nucleotide) upstream and downstream sequence contexts were used to calculate the cG/cC score, and 2.05 was taken as the threshold for the preliminary inference of the G-quadruplex folding capability (Supplementary Table 4) (Beaudoin et al., 2014). Using a customized python script, we implemented the cG/cC scoring system, and the source code of the python script could be found at GitHub.

### Homo-Dimer Homology Modeling and Electrostatic Potential Calculation

The homo-dimer of the SUD-homology in SARS-CoV-2 was modeled based on the template of the SARS-CoV SUD structure (PDB ID: 2W2G) through homology modeling. All the modeling process was performed in the Swiss Model (Waterhouse et al., 2018) website (https://swissmodel.expasy.org/) according to the default options. The electrostatic potential was calculated and visualized in the PyMOL software by using the APBS (adaptive Poisson–Boltzmann solver) plugin under the default parameters.

### ΔG° Z-score Analysis

The ΔG° z-score for the SARS-CoV-2 genome was retrieved from RNAStructuromeDB (https://structurome.bb.iastate.edu/sars-cov-2) (Andrews et al., 2017). The ΔG° z-score is described as follows (Andrews et al., 2020).

(1) $$\begin{array}{l} \Delta G^{{}^{\circ}}z-score=\frac{\left(MFE_{native}-{\bar{MFE}}_{random}\right)}{\sigma } \end{array}$$

where the *MFE*_*native*_ means the MFE (minimum free energy) ΔG° value predicted by the RNAfold software with a window of 120 nt and step of 1 nt. In addition, the ${\bar{MFE}}_{random}$ represents the MFE ΔG° value generated by the randomly shuffled sequence with the identical nucleotide composition. The σ is the standard deviation across all the MFE values.

To depict the ΔG° z-score for each nucleotide in the SARS-CoV-2 genome, we utilized the following formula.

(2) $$\begin{array}{l} {\text{z}}_{i}=\frac{\sum_{m=1}^{w}{\Delta {\text{G}}^{{}^{\circ}}z-\text{score}}_{m}}{w} \end{array}$$

where z_*i*_ is the average ΔG° z-score for nucleotide *i*, and *w* denotes the total number of the sliding windows that cover the nucleotide *i*. ${\Delta \text{G}{\text{z}}^{{}^{\circ}}-\text{score}}_{m}$ indicates the ΔG° z-score for the m-th window. For example, when considering the nucleotide 1,000 under the setting of 120 nt window length and 1-nt step, there are 120 sliding windows covering the nucleotide 1,000. So, the *z*_200_, which means the average ΔG° z-score for nucleotide 200, is calculated as the sum of the ΔG° z-score of 120 sliding windows divided by the total number of the sliding windows.

## Results

### Whole Genome Identification and Annotation of Potential G-Quadruplexes

**Figure 2:**
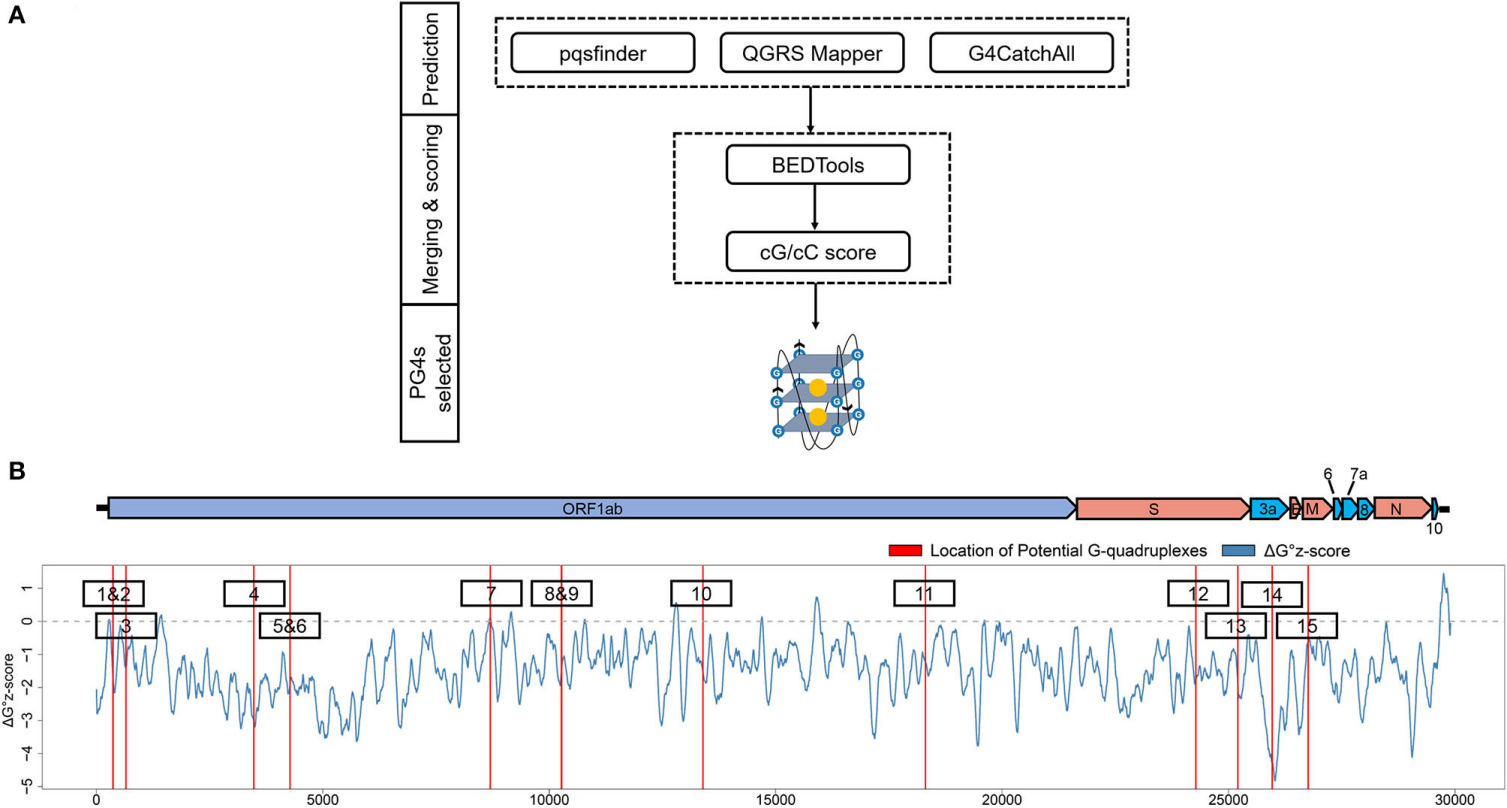
Detection and annotation of the potential G-quadruplexes (PG4s). **(A)** The schematic flow of PG4s detection. The G-quadruplex prediction tools, pqsfinder, QGRS Mapper, and G4CatchAll were utilized for the prediction of PG4s. BEDTools and the cG/cC scoring system were applied to merge and score the PG4s. After screening, the PG4s that were used in this study were generated. **(B)** Visualization of the PG4s in the SARS-CoV-2 genome. Top: the genome organization of SARS-CoV-2. Bottom: the average ΔG° z-score for each nucleotide (blue curve) in the SARS-CoV-2 genome; the location of PG4s are plotted with red vertical lines. The order of PG4s is marked with a black box. Please note that only the PG4s in the positive-sense strand are visualized.

To get the potential G-quadruplexes in SARS-CoV-2, we took the strategy described as follows (Figure 2A): (i) Predicting the PG4s with three software independently. (ii) Merging the prediction results of the PG4s and evaluating the G-quadruplex folding capabilities by the cG/cC scores. (iii) The PG4s with cG/cC scores higher than the threshold were selected as candidates for further analysis. Here, the threshold for determining whether PG4s can be folded was set to 2.05, as described in the study of Beaudoin et al. (2014). In total, we obtained 24 PG4s (Table 1) in the positive- and negative-sense strands for further analysis.

**Table 1:** The potential G-quadruplexes (PG4s) found in severe acute respiratory syndrome coronavirus 2 (SARS-CoV-2).

**No**.	**Start**	**End**	**Strand**	**Sequence (5^**′**^ → 3^**′**^)**	**Annotation**
1	15	37	–	GGTTGGTTTGTTACCTGGGAAGG	–
2	353	377	+	GGCTTTGGAGACTCCGTGGAGGAGG	nsp1
3	359	377	+	GGAGACTCCGTGGAGGAGG	nsp1
4	644	663	+	GGTAATAAAGGAGCTGGTGG	nsp1
5	2,449	2,472	–	GGGGCTTTTAGAGGCATGAGTAGG	–
6	3,467	3,483	+	GGAGGAGGTGTTGCAGG	nsp3
7	4,261	4,289	+	GGGTTTAAATGGTTACACTGTAGAGGAGG	nsp3
8	4,262	4,289	+	GGTTTAAATGGTTACACTGTAGAGGAGG	nsp3
9	4,886	4,901	–	GGTGGAATGTGGTAGG	–
10	6,011	6,027	–	GGATATGGTTGGTTTGG	–
11	8,687	8,709	+	GGATACAAGGCTATTGATGGTGG	nsp4
12	10,015	10,030	–	GGTTTGTGGTGGTTGG	–
13	10,015	10,039	–	GGTGATAGAGGTTTGTGGTGGTTGG	–
14	10,019	10,039	–	GGTGATAGAGGTTTGTGGTGG	–
15	10,255	10,282	+	GGTACAGGCTGGTAATGTTCAACTCAGG	nsp5
16	10,261	10,290	+	GGCTGGTAATGTTCAACTCAGGGTTATTGG	nsp5
17	13,385	13,404	+	GGTATGTGGAAAGGTTATGG	nsp10
18	15,924	15,941	–	GGATCTGGGTAAGGAAGG	–
19	18,296	18,318	+	GGATTGGCTTCGATGTCGAGGGG	nsp14
20	24,268	24,291	+	GGCTTATAGGTTTAATGGTATTGG	S-S2
21	25,197	25,218	+	GGCCATGGTACATTTGGCTAGG	S-S2
22	25,951	25,979	+	GGTGGTTATACTGAAAAATGGGAATCTGG	ORF3a
23	26,746	26,775	+	GGATCACCGGTGGAATTGCTATCGCAATGG	M
24	26,889	26,917	–	GGTCTGGTCAGAATAGTGCCATGGAGTGG	–

To annotate the PG4s, the reference annotation data (in gff3 format) of SARS-CoV-2 were downloaded from the NCBI database with the accession number of NC_045512. First, we focused on the PG4s in the positive-sense strand. Fifteen of the 24 PG4s (67.5%) were located in the positive-sense strand (Table 1); most of them were harbored in non-structural proteins including nsp1, nsp3, nsp4, nsp5, nsp10, and nsp14, with the remaining ones located in the spike protein, orf3a, and the membrane protein. Second, we examined the PG4s in the negative-sense strand, which is an intermediate product of replication. Nine PG4s were scattered in the negative-sense strand (Table 1).

To further characterize the potential canonical secondary structures competitive with G-quadruplexes, the landscape of thermodynamic stability of the SARS-CoV-2 genome was depicted by using ΔG° z-score (Andrews et al., 2020). In general, a positive ΔG° z-score implies that the secondary structure of this region tends to be less stable than the randomly shuffled sequence with the identical nucleotide composition, while a negative ΔG° z-score signifies higher stability than the randomly shuffled sequence. For each nucleotide in the SARS-CoV-2 genome, the ΔG° z-score was calculated for all the 120 nt windows covering the nucleotide, and an average ΔG° z-score was deduced then. Several PG4s are located in positions with a locally higher average ΔG° z-scores (Figure 2B) which implied the relative instability of a canonical secondary structure and the lower possibility to adopt such a competitive structure against the G-quadruplex structure, which may ultimately favor the formation of G-quadruplex structure.

### Potential G-Quadruplexes in SARS-CoV-2 Show Analogical Features With the rG4s in the Human Transcriptome

In 2016, Chun Kit Kwok and coworkers profiled the RNA G-quadruplexes in the HeLa transcriptome by using the RNA G-quadruplex sequencing (rG4-seq) technology, and quantified the diversity of these RNA G-quadruplexes (Kwok et al., 2016). We set out to address the question of whether the potential G-quadruplexes in SARS-CoV-2 showed analogical features with the G-quadruplexes found in the human transcriptome and if these PG4s have the ability to form G-quadruplex structures. We noticed that the PG4s in SARS-CoV-2 are all in the two-quartet style. Therefore, we retrieved the two-quartet RNA G-quadruplex sequence data generated in the rG4-seq experiment under the condition of K+ and pyridostatin (PDS). However, for some RTS (reverse transcriptase stalling) sites labeled as two-quartet, there may exist overlapping G-quadruplexes with different loops (e.g., GGCACAGCAGGCATCGGAGGTGAGGCGGGG), and it is difficult to determine which one was formed in the experiment. In order to eliminate the ambiguity, only the RTS sites containing non-overlapping two-quartet G-quadruplex (e.g., GTCATTTTTTGTGTTTGGTTTGGTGGTGGC) were considered.

**Figure 3:**
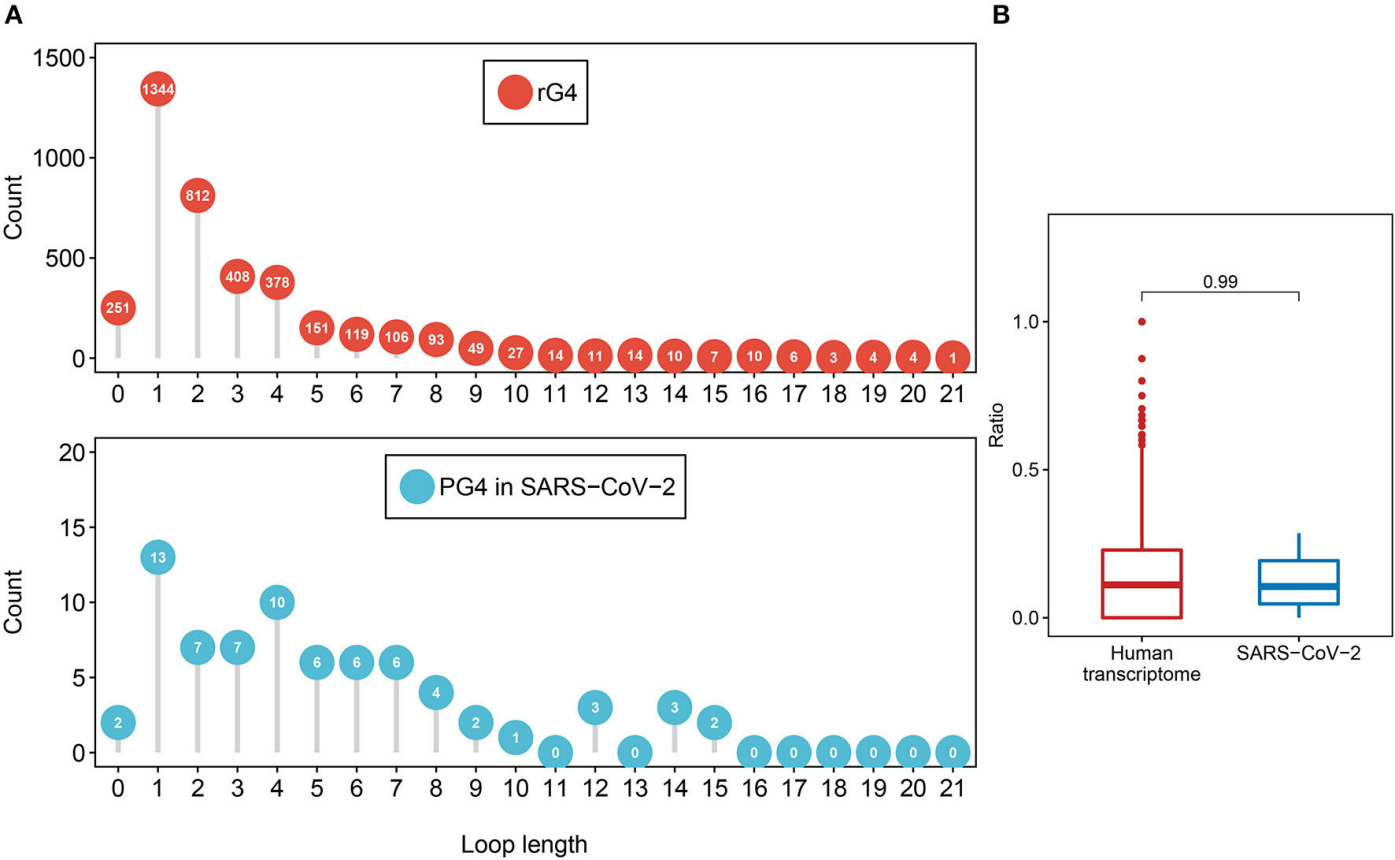
Feature comparison of potential G-quadruplexes found in rG4-seq and SARS-CoV-2. **(A)** The dot chart represents the number of two-quartet G-quadruplex loops with different lengths in the human transcriptome and SARS-CoV-2, respectively. Top: G-quadruplex in the human transcriptome. Bottom: G-quadruple in SARS-CoV-2. **(B)** The red boxplot shows the ratio of cytosine in the human transcriptome two-quartet G-quadruplex loops, and the blue boxplot displays the ratio of cytosine in the SARS-CoV-2 PG4 loops.

First, we investigated the loop length distribution pattern of the two-quartet PG4s in both SARS-CoV-2 and the human transcriptome (Figure 3A). As a whole, the two-quartet PG4s in SARS-CoV-2 and the human transcriptome displayed similar loop length distribution patterns, and the loop length of the PG4s in SARS-CoV-2 falls into the scope of the ones from the human transcriptome. The distributions of loop length between the SARS-CoV-2 PG4s and the human two-quartet G-quadruplexes did not show discrepancies (Supplementary Figure 1, Wilcoxon test, *p* = 0.4552).

Considering the fact that the presence of multiple cytosine tracks may hinder the formation of G-quadruplex structures (Beaudoin, 2010; Beaudoin et al., 2014), we examined the cytosine ratio in G-quadruplex loops (Figure 3B). No significant difference in loop cytosine ratios was observed between the SARS-CoV-2 PG4s and the human two-quartet G-quadruplexes (Wilcoxon test, *p* = 0.9911), which suggested that the loop cytosine ratios between the two types of G-quadruplex were similar.

Taken together, our results suggested that the PG4s in SARS-CoV-2 displayed similar features to the rG4s in the human transcriptome.

### Potential G-Quadruplexes Are Under Heightened Selective Constraints in Bat- and Pangolin-Related Betacoronaviruses

Recent research revealed that the G-quadruplexes in human UTRs (untranslated regions) are under selective pressures (Lee et al., 2020), and some coronaviruses on bats and pangolins are closely related to SARS-CoV-2 (Lam et al., 2020; Zhang T. et al., 2020). Consequently, we wondered whether the potential G-quadruplexes in the SARS-CoV-2 genome are under heightened selective constraints. We collected some betacoronavirus genomic sequences of bats and pangolins from several public databases and used the NJ (neighbor-joining) method to construct the phylogenetic tree with 1,000 bootstrap replications (Supplementary Figure 2). The RS (rejected substitutions) score for each site in the SARS-CoV-2 reference genome was evaluated by using the GERP++ software.

**Figure 4:**
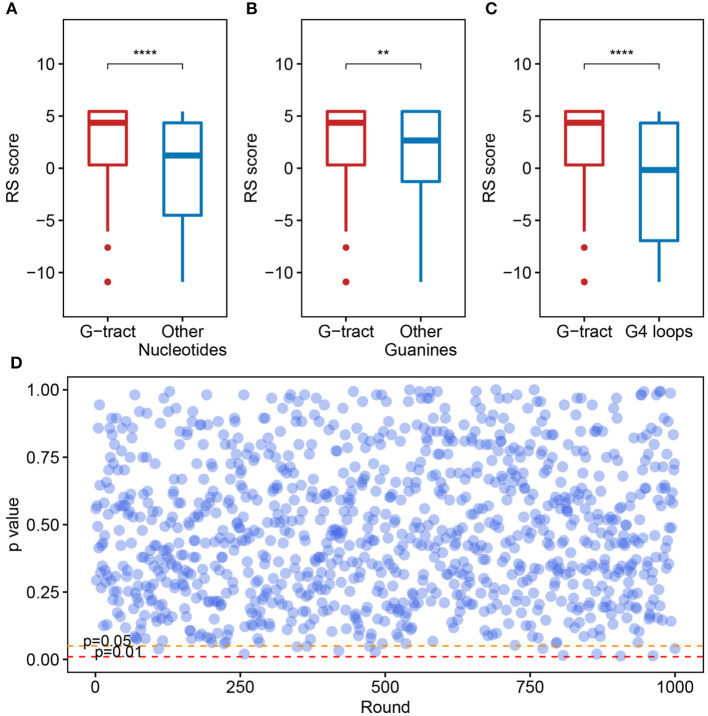
Potential G-quadruplexes exhibit heightened selective constraints in bat and pangolin related betacoronavirus. **(A–C)** Boxplot showing the difference of nucleotide RS scores in G-tract, other nucleotides, other guanines, and PG4 loops (**p ≤ 0.01, ****p ≤ 0.0001). **(D)** Test of the RS score difference between the fragments containing PG4s and the randomly selected fragments. The abscissa indicates the round of the test, while the ordinate represents the *p*-value for each round.

We checked the RS score difference between the G-tract (continuous runs of G) nucleotides and other nucleotides. A significant discrepancy was observed, which means that the G-tract nucleotides exhibit heightened selective constraints than other nucleotides in the SARS-CoV-2 genome (Figure 4A, Wilcoxon test, *p* = 9.254 × 10^−8^). Considering that the G-tracts are composed of guanines, the conservation of guanines in and outside the G-tracts in the SARS-CoV-2 genome were also compared. We discovered that the guanines in G-tracts are under heightened selective constraints (Figure 4B, Wilcoxon test, *p* = 3.363 × 10^−3^). The nucleotides within G-tracts are more relevant to the G-quadruplex structural maintenance than the loops. Then we compared the G-tract and loop RS scores. As a result, the G-tract RS scores were significantly higher than that of the loops (Figure 4C, Wilcoxon test, *p* = 3.962 × 10^−7^), which suggests that the G-tracts experienced stronger selective constraints.

We also checked if the PG4s that are under heightened selective constraints is relevant to its inherent properties rather than the sequence contexts. A random test was performed to check whether the fragments containing PG4s manifested different average RS scores compared with random fragments in the SARS-CoV-2 genome. The fragments containing PG4s were designated as the sequence 100 nt upstream and downstream of the PG4 centers. We conducted 1,000 rounds of tests. In each test, we randomly selected 50 fragments from the SARS-CoV-2 genome with a length of 200 nt and carried out the Wilcoxon test to assess the average RS score difference among the randomly selected fragments and the fragments containing PG4s. The *p*-value for each round was retained. As a result, no evident difference was observed as few *p*-values (13/1,000) were < 0.05 (Figure 4D), suggesting that PG4s that are under heightened selective constraints are more likely to be related to its inherent properties rather than sequence contexts.

### SARS-CoV-2 Contains Similar SUD to SARS-CoV

**Figure 5:**
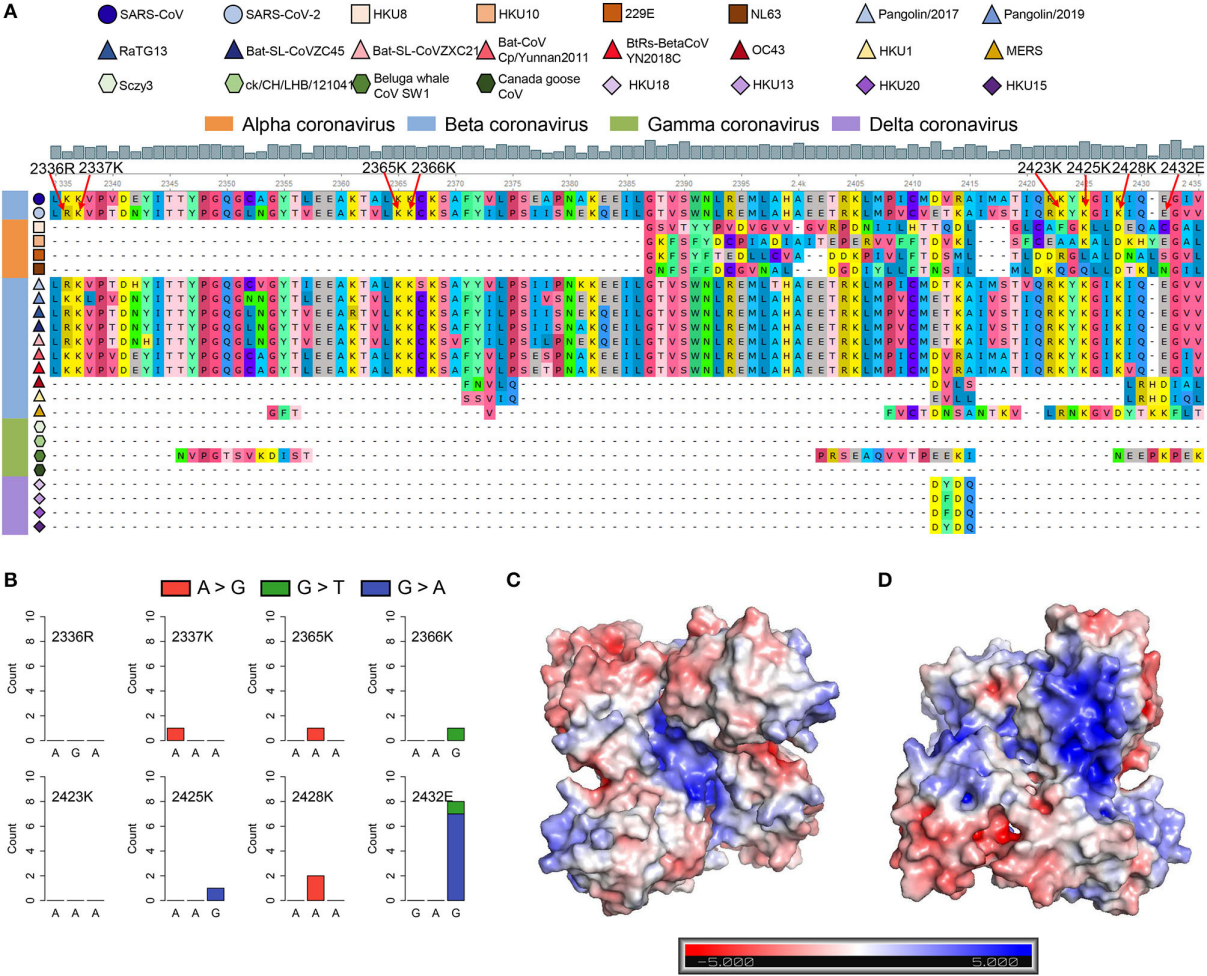
SARS-CoV-2 contains the SARS-unique domain (SUD)-homology sequence and dimer structure. **(A)** Sequence alignment of four different genera coronaviruses. The shapes in various colors mark different kinds of coronaviruses. The color bar represents different genera of coronaviruses (orange, alphacoronavirus; blue, betacoronavirus; green, gammacoronavirus; purple, deltacoronavirus). The gray histogram shows the consensus of the alignment sites. The eight amino acid residues related to the G-quadruplex binding affinity are labeled by red arrows. **(B)** Nucleotide mutation count of eight amino acid residues among 16,466 SARS-CoV-2 samples. **(C,D)** The electrostatic potential surface of the SARS-CoV-2 SUD-homology dimer with different orientations [the front **(C)** and rotate 180° **(D)**]. The blue and red showed positive and negative potentials, respectively. The positively charged patches in the center **(C)** and back **(D)** of the SUD-homology dimer might be the binding domain for G-quadruplexes.

SARS-CoV and SARS-CoV-2 share similar nucleic acid sequence compositions, and both can cause acute disease symptoms. It has been confirmed that the SUD in the SARS-CoV could bind to the G-quadruplex structures in human transcripts (Tan et al., 2009), but whether SARS-CoV-2 possesses a similar SUD structure remains unclear. Thus, we explored whether the SARS-CoV-2 genome contains the protein-coding sequence potentially encoding SUD-homology and whether SARS-CoV-2 retains the ability to bind RNA G-quadruplex structures. We collected the ORF1ab amino acid sequences of some coronaviruses belonging to different genera, including seven known coronaviruses that can infect humans. The SUD-homology amino acid sequence was absent in some coronaviruses, especially in alpha, gamma, and delta coronaviruses (Supplementary Figure 3). In contrast, the SUD-homology sequence was retained in several betacoronavirus, particularly in bat- and pangolin-associated ones. Moreover, among the seven coronaviruses that can infect humans, only SARS-CoV and SARS-CoV-2 kept the SUD or SUD-homology sequence, while the sequence in MERS-CoV, HCoV-229E, HCoV-NL63, HCoV-OC43, and HCoV-HKU1 were depleted. Next, we examined the eight key amino acid residues in SUD that are related to G-quadruplex binding affinity according to the previous reports (Figure 5A). Almost all the key amino acid residues are reserved in SARS-CoV-2, with one exception of a conservative replacement of K (Lysine) with R (Arginine). The conservation of the eight amino acid residues within SARS-CoV-2 samples was then investigated, to see if these residues were conserved and the G-quadruplex binding ability of SUD-homology was essential for SARS-CoV-2. We retrieved the nucleotide sequence alignment file of 16,466 SARS-CoV-2 samples from the GISAID database and calculated the mutation frequency for each nucleotide. As a result, limited mutation frequencies were found in the eight amino acid residues compared to the whole genome average mutation frequency (Figure 5B, frequency = 3.96). Although eight nucleotide mutations were detected of glutamate (2,432 E), seven of them were synonymous mutations. Next, we checked the electrostatic potential pattern of the SUD-homology dimer from SARS-CoV-2. The positively charged patches were observed in the core of the SUD-homology dimer, which was surrounded by negatively charged patches (Figure 5C). In contrast, when the dimer was rotated 180°, a slightly inclined narrow cleft with negative potential accompanied by the positively charged patches was discovered (Figure 5D). The above results indicated that the SUD-homology dimer of SARS-CoV-2 and the SUD_core_ (a shortened version of SUD) dimer of SARS presented analogical electrostatic potential patterns [see ref (Tan et al., 2009) for more details about the electrostatic potential surface of the SUD_core_ homodimer in SARS]. Similar to the SUD dimer of SARS, we identified the positively charged patches located in the center and back of the SARS-CoV-2 SUD-homology dimer that can potentially bind the G-quadruplex structures (Figures 5C,D).

## Discussion

The COVID-19 pandemic has caused huge losses to humans and made people pay more attention to public health. A large number of scientists around the world have participated in the fight against the epidemic. The SARS-CoV-2 coronavirus is the key culprit responsible for the outbreak, and no specific inhibitor drugs have been developed yet. G-quadruplexes have shown tremendous potential for the development of anticancer (Han and Hurley, 2000; Balasubramanian et al., 2011; Miller and Rodriguez, 2011; Neidle, 2017) and antiviral drugs (Perrone et al., 2015; Ruggiero and Richter, 2018, 2020), as G-quadruplexes can interfere with many biological processes that are critical to cancer cells and viruses. Therefore, it is necessary to quantify and characterize the PG4s in the SARS-CoV-2 genome to provide a possible novel method for the treatment of COVID-19.

In this study, besides three popular G-quadruplexes prediction tools, the cG/cC scoring system, which is specially designed for the identification of RNA G-quadruplexes, was adopted to determine the PG4s. Indeed, we did not find the G-quadruplexes with three or more G-quartets, which are generally considered to be more stable than the two-quartet G-quadruplexes. One of the controversial issues lies on the stability of the two-quartet G-quadruplexes, especially the folding capability of those G-quadruplexes *in vivo*. However, it is well-acknowledged that the RNA G-quadruplexes is more stable than their DNA counterparts (Joachimi et al., 2009; Zaccaria and Fonseca Guerra, 2018) and SARS-CoV-2 is a single-strand RNA virus, which may be conducive to its structure formation. Several emerging studies have demonstrated the formation of two-quartet G-quadruplexes in viral sequences (Perrone et al., 2013; Murat et al., 2014; Fleming et al., 2016; Wang et al., 2016a; Zahin et al., 2018; Majee et al., 2020; Zhang Y. et al., 2020), which could serve as antiviral elements under the presence of G-quadruplex ligands. We also did a comprehensive search for viral two-quartet G-quadruplexes, whose formation has been confirmed by experimental methods *in vitro*, and a total of 16 two-quartet G-quadruplexes were found (Supplementary Table 5). The potential G-quadruplexes in SARS-CoV-2 showed a lower cytosine ratio in loops than that in experimental validated viral G-quadruplexes (Supplementary Figure 4A, Wilcoxon test, *p* = 0.0018), which is a positive signal for the G-quadruplex formation in SARS-CoV-2. When looking at the loop lengths, the experimental supported G-quadruplexes in other viruses displayed a short and concentrated distribution mode, while the potential G-quadruplexes in SARS-CoV-2 exhibited a relatively uniform distribution mode in <8 nt, which is a hint that maybe part of the sequence could form G-quadruplex structures (Supplementary Figure 4B). The cG/cC scores of the experimental supported G-quadruplexes in other viruses and their 15 nt flanking sequences were calculated as well (Supplementary Table 6). The cG/cC scores of some potential G-quadruplexes in SARS-CoV-2 were relatively high (Supplementary Figure 5); however, the overall cG/cC scores for the potential G-quadruplexes in SARS-CoV-2 were low. When only considering the G-quadruplex sequences itself, the potential G-quadruplexes in SARS-CoV-2 presented higher cG/cC scores (mean = 52.51, standard deviation = 77.29), while the experimental supported G-quadruplexes displayed relatively lower cG/cC scores (mean = 24.39, standard deviation = 37.27). These observations suggest that some of the potential G-quadruplexes in SARS-CoV-2 could be folded into secondary structures. Moreover, the K^+^ (potassium ion), one of the primary positive ions inside human cells, can strongly support the formation of G-quadruplexes. Nevertheless, whether the SARS-CoV-2 G-quadruplexes could form *in vivo* requires overwhelming proofs.

Most of the PG4s we detected were located in the positive-sense strand. The G-quadruplex forming sequences in the SARS-CoV genome were presumed to function as the chaperones of SUD, and their interaction was essential for the SARS-CoV genome replication (Kusov et al., 2015). ORF1ab that encodes the replicase proteins is required for the viral replication and transcription. Some PG4s were found to harbor in ORF1ab, and whether these PG4s were related to the replication of the viral genome and interacted with the G-quadruplex binding domain in SARS-CoV-2 is worthy of further investigation. In addition to ORF1ab, there exist several PG4s in the structural and accessory protein-coding sequences as well as the sgRNAs that contain the above protein sequences. Some studies have characterized the impact of G-quadruplex structures on the translation of human transcripts, and an apparent inhibitory effect was observed (Kumari et al., 2007; Beaudoin, 2010; Shahid et al., 2010). The translation of some SARS-CoV-2 proteins requires the involvement of human ribosomes; thus, it is possible to repress the translation of SARS-CoV-2 proteins via stabilizing the G-quadruplex structures. In fact, this inhibition effect has been reported in some other viral studies (Wang et al., 2016b; Majee et al., 2020). The negative-sense strand serves as templates for the synthesis of the positive-sense strand and the subgenomic RNAs. The identified potential G-quadruplexes were broadly distributed in the negative-sense strand of SARS-CoV-2. Notably, we observed one PG4 located at the 3′ end of the negative-sense strand. A previous study confirmed that the stable G-quadruplex structures located at the 3′ end of the hepatitis C virus negative-sense strand could inhibit the RNA synthesis by reducing the activity of the RdRp (RNA-dependent RNA polymerase) (Jaubert et al., 2018). Therefore, it is necessary to further investigate whether the PG4 at the 3′ end of the negative-sense strand of SARS-CoV-2 could inhibit RNA synthesis. In addition, recent studies have detected high-frequency trinucleotide mutations (G28881A, G2882A, and G28883C) in the SARS-CoV-2 genome (Yao et al., 2020; Yin, 2020). G28881A and G28882A always co-occur within the same codon, which means a positive selection of amino acid (Mishra et al., 2020), but the consequence of the trinucleotide mutations was still elusive. We noticed that the trinucleotide mutations were in the G-rich sequence from 28,881 to 28,917 nt (5′ GGGGAACTTCTCCTGCTAGAATGG**CT**GG**CAAT**GG**C**GG 3′). The potential G-quadruplex downstream of the trinucleotide mutations was filtered by the cG/cC score system as the presence of cytosine tracks within and flanking of the potential G-quadruplex reduce the cG/cC score; however, in fact, this potential G-quadruplex showed a relative lower MFE (Minimum Free Energy) among all the potential G-quadruplexes we detected. Whether the mutations have an internal causality with the G-rich sequence still needs to be elucidated.

The SUD in SARS, which is thought to be related to its terrible pathogenicity, has displayed binding preference to the G-quadruplexes in human transcripts (Tan et al., 2009). Our analysis revealed that the novel coronavirus SARS-CoV-2 contained a similar domain to SUD as well. Furthermore, several amino acid residues previously reported to be an indispensable part of the G-quadruplexes binding capability are almost retained in SARS-CoV-2. Further exploration indicated that the eight key amino acid residues were conserved in numerous SARS-CoV-2 samples across countries all over the world, suggesting the essentiality of the above residues. It is supposed that the binding of SUD to G-quadruplexes could affect transcript stability and translation, hence, impairing the immune response of host cells (Tan et al., 2009). The expression of host genes in SARS-CoV-2-infected cells is extremely inhibited (Kim et al., 2020); therefore, we speculate that the SARS-CoV-2 may possess a similar mechanism to SARS-CoV that can inhibit the expression of some important genes.

**Figure 6:**
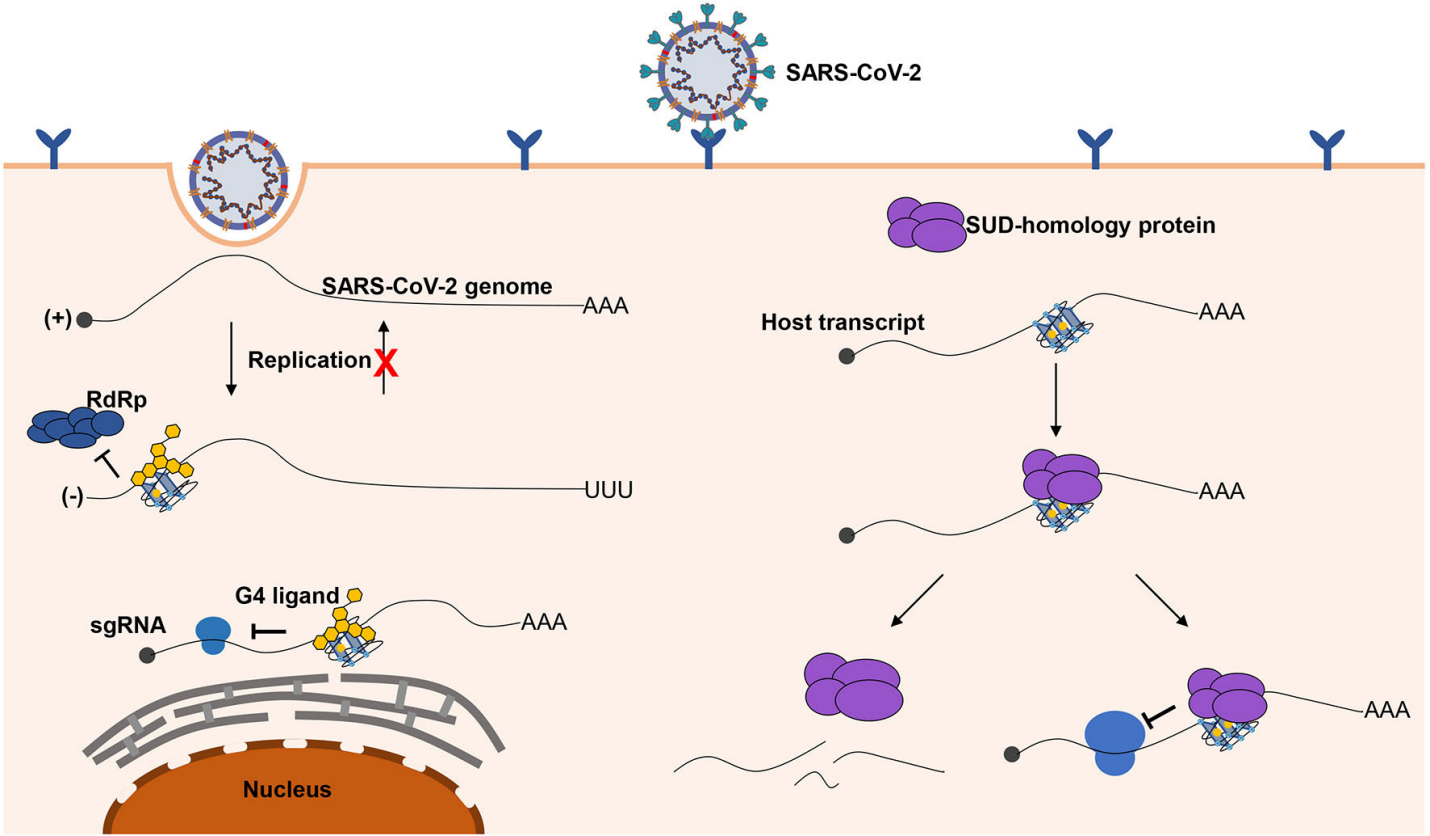
Possible role of G-quadruplexes in the antiviral mechanism and pathogenicity. Left part, G-quadruplexes can function as inhibition elements in the SARS-CoV-2 life cycle. Both the replication and translation could be affected by the G-quadruplex structures. The stable G-quadruplex structures in the 3′ end of the negative-sense strand may interfere with the activity of RdRp; hence, the replication of the negative-sense strands to the positive-sense strands is repressed, so that the SARS-CoV-2 genomes cannot be produced in large quantities. The G-quadruplex structures can suppress the translation process by impairing the elongating of ribosomes, which can hinder the production of proteins required for the virus. The G-quadruplex structures could be stabilized by the specific ligands to enhance the inhibitory effects, which is a promising antiviral strategy. Right part, a possible mechanism for SARS-CoV-2 to impede the expression of human genes. G-quadruplex structures, particularly with longer G-stretches, are the potential binding targets for the G-quadruplex binding domain in SARS-CoV-2, and the interaction of the G-quadruplex binding domain of SARS-CoV-2 with G-quadruplex structures possibly leads to the transcript instability or obstructing of the translation efficiency.

Herein, we briefly depict the possible role of G-quadruplexes in the antiviral mechanism and pathogenicity, and the development of certain G-quadruplex-specific ligands might be a promising antiviral strategy (Figure 6). We call for more researchers to shed light on the relationship between G-quadruplexes and coronaviruses. Only if we have a deeper understanding of coronaviruses can we better cope with the possible novel coronavirus pandemics in the future.

## Data Availability Statement

The datasets generated for this study can be found in online repositories. The names of the repository/repositories and accession number(s) can be found in the article/Supplementary Material.

## Author Contributions

This project was under the supervision of XS. RZ, KX, and YG took part in the project. The article was written by RZ and revised by XS and KX. HL participated in the discussion of this project. All authors contributed to the article and approved the submitted version.

## Conflict of Interest

The authors declare that the research was conducted in the absence of any commercial or financial relationships that could be construed as a potential conflict of interest.
