# Supplementary material for: Whole Genome Identification of Potential G-Quadruplexes and Analysis of the G-Quadruplex Binding Domain for SARS-CoV-2
Source: Front Genet. 2020 Nov 27;11:587829. doi: 10.3389/fgene.2020.587829 (PMC7728997; doi:10.3389/fgene.2020.587829)
Supplement: Supplementary file 1 [file Data_Sheet_1.docx]

Supplementary Material

Whole genome identification of potential G-quadruplexes and analysis of the G-quadruplex binding domain for SARS-CoV-2

Rongxin Zhang^1^, Ke Xiao^1^, Yu Gu^1^, Hongde Liu^1^, Xiao Sun^1*^

^1^State Key Laboratory of Bioelectronics, School of Biological Science and Medical Engineering, Southeast University, Nanjing, China

*** Correspondence:**Xiao Sun
xsun@seu.edu.cn

## Supplementary Figures


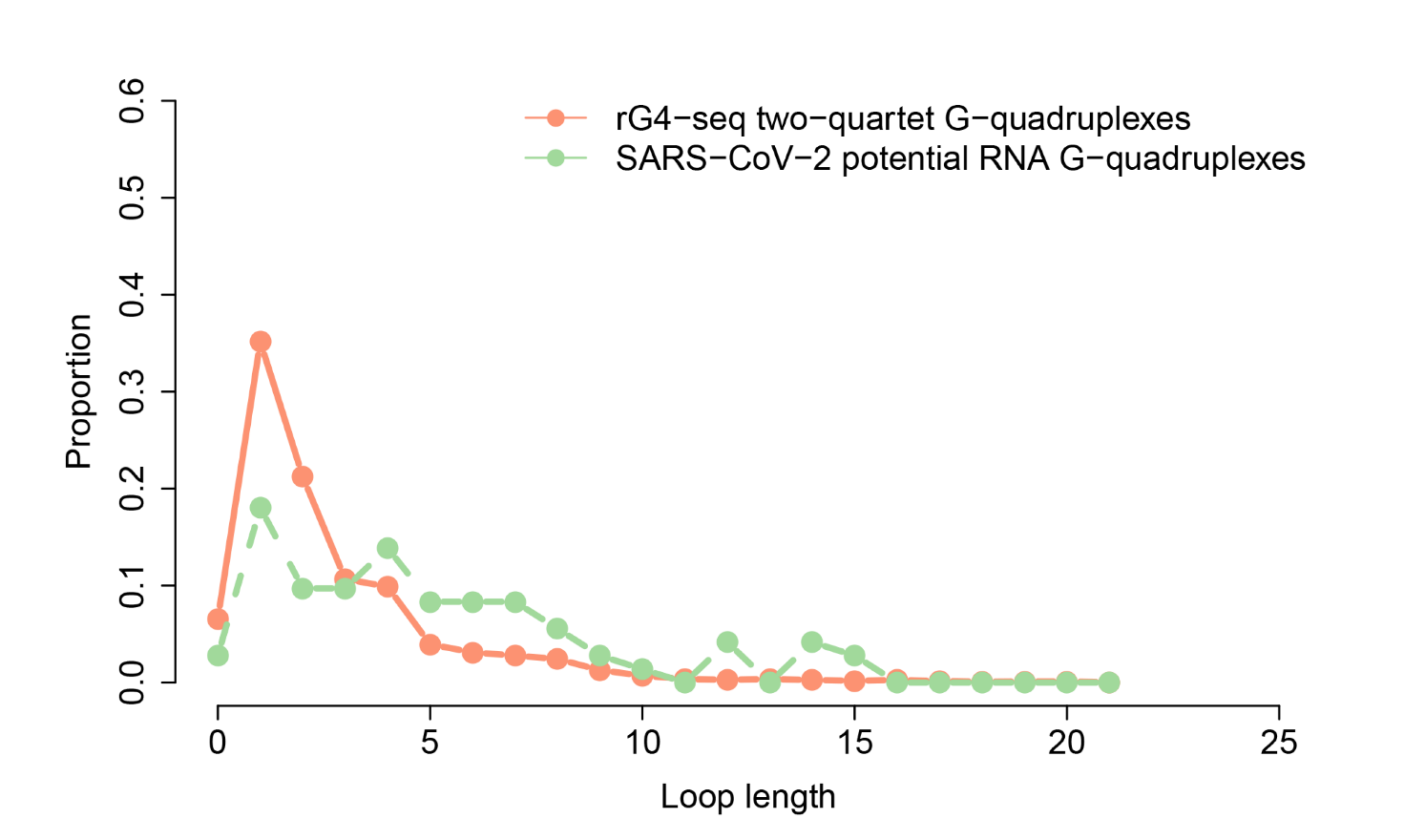


Supplementary Figure 1. Proportion of loops with different lengths. The x-axis and y-axis show the loop length and loop proportion, respectively. The green curve represents the loop in SARS-CoV-2 PG4s, while the red curve indicates the loop in two-quartet G-quadruplexes derived from rG4-seq.


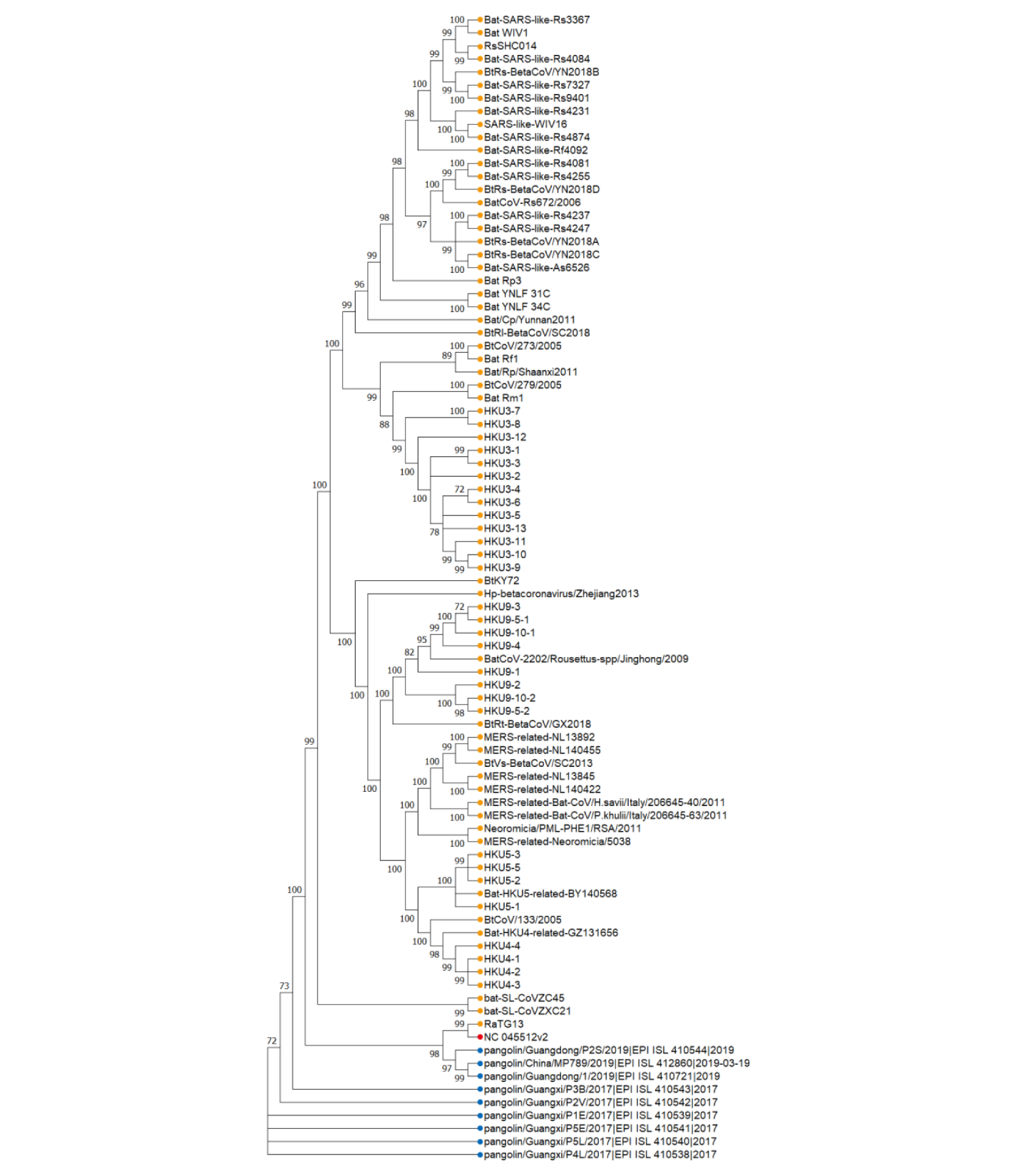


Supplementary Figure 2. Phylogenetic tree of bat and pangolin related betacoronavirus. The phylogenetic tree was constructed using the Neighbor-Joining method with 1,000 bootstrap replications. The bootstrap values lower than 70 were removed from the phylogenetic tree nodes. The SARS-CoV-2 reference sample is marked in the red dot, and the orange dots indicate the bat-related betacoronavirus samples, while the blue dots represent pangolin related betacoronavirus samples.


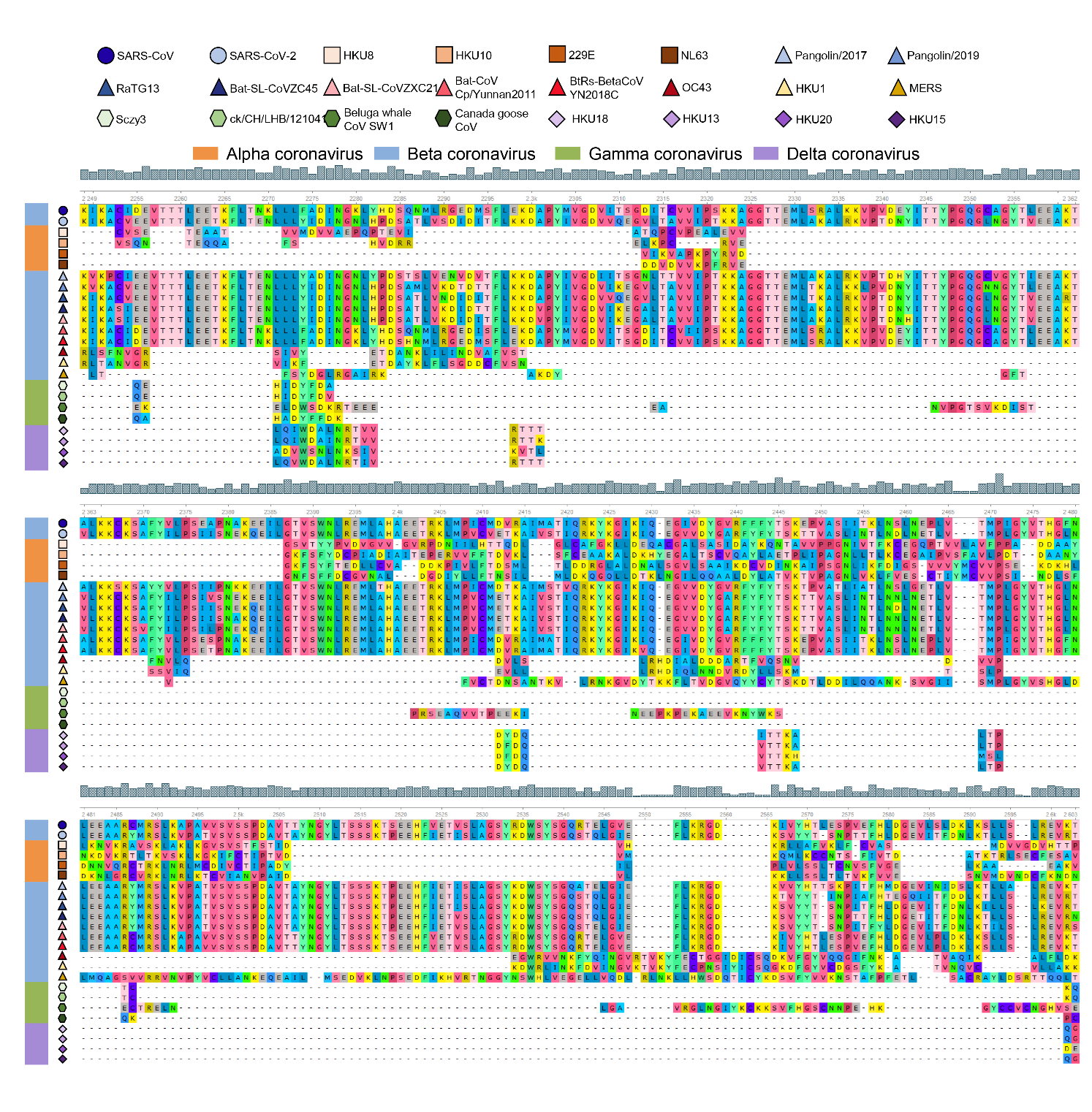


Supplementary Figure 3. Alignment of several coronavirus amino acid sequences. The figure shows the sequence alignment corresponding to SARS SUD. The shapes in various colors mark different kinds of coronaviruses. The color bar represents different genera of coronaviruses (orange, alphacoronavirus; blue, betacoronavirus; green, gammacoronavirus; purple, deltacoronavirus). The grey histogram shows the consensus of the alignment sites.


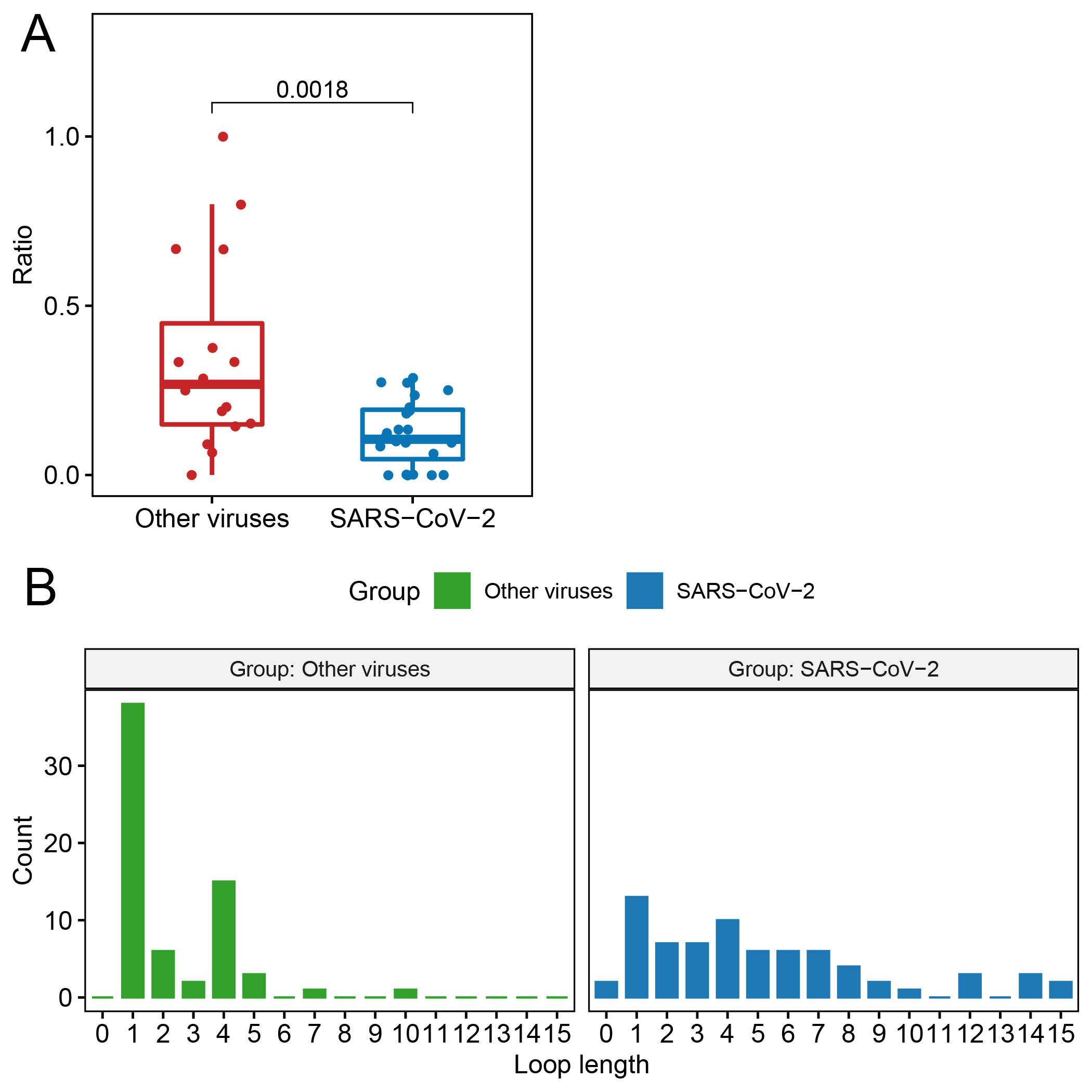


Supplementary Figure 4. Comparison of cytosine ratio and length in G-quadruplex loops between SARS-CoV-2 and other viruses. (A) Cytosine ratio in G-quadruplex loops. The red box represents the cytosine ratio of loops in the G-quadruplexes of other viruses supported by chemical or biological experiments; the blue box indicates the cytosine ratio of loops in the potential G-quadruplex of SARS-CoV-2. (B) Count of different loop lengths for G-quadruplexes. The green and blue bars designate the G-quadruplex in other viruses and SARS-CoV-2, respectively.


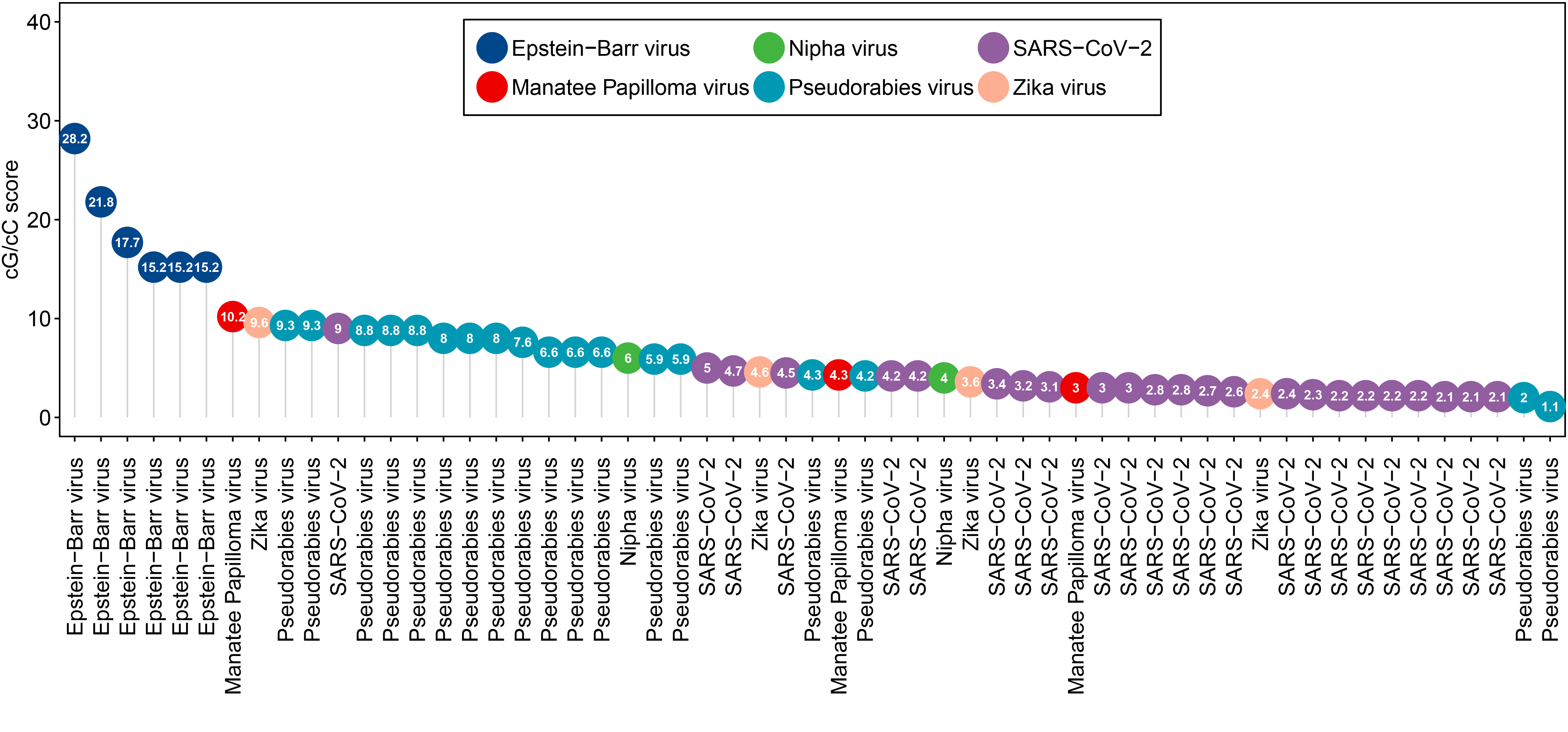


Supplementary Figure 5. cG/cC score for the potential G-quadruplexes in SARS-CoV-2 and the experimental supported G-quadruplexes in other viruses. Each dot represents a cG/cC score for a specific G-quadruplex. Please note, for a particular G-quadruplex in Epstein-Barr or pseudorabies virus, it may appear multiple times in the genome of the aforementioned virus.

## Supplementary Tables

Supplementary Table 1. Nucleic acid sequence information of the viruses used in this study. Due to the extremely large number of SARS-CoV-2 sample sequences retrieved from GISAID database, the detailed 16,466 SARS-CoV-2 sample information is not provided.

| **No.** | **Accession number** | **Virus** | **Source** |
| --- | --- | --- | --- |
| 1 | DQ648794 | Bat coronavirus (BtCoV/133/2005) | DBatVir/Genebank |
| 2 | JX993988 | Bat coronavirus Cp/Yunnan2011 | DBatVir/Genebank |
| 3 | EF065505 | Bat coronavirus HKU4-1 | DBatVir/Genebank |
| 4 | EF065506 | Bat coronavirus HKU4-2 | DBatVir/Genebank |
| 5 | EF065507 | Bat coronavirus HKU4-3 | DBatVir/Genebank |
| 6 | EF065508 | Bat coronavirus HKU4-4 | DBatVir/Genebank |
| 7 | EF065509 | Bat coronavirus HKU5-1 | DBatVir/Genebank |
| 8 | EF065510 | Bat coronavirus HKU5-2 | DBatVir/Genebank |
| 9 | EF065511 | Bat coronavirus HKU5-3 | DBatVir/Genebank |
| 10 | EF065512 | Bat coronavirus HKU5-5 | DBatVir/Genebank |
| 11 | EF065513 | Bat coronavirus HKU9-1 | DBatVir/Genebank |
| 12 | HM211100 | Bat coronavirus HKU9-10-1 | DBatVir/Genebank |
| 13 | HM211101 | Bat coronavirus HKU9-10-2 | DBatVir/Genebank |
| 14 | EF065514 | Bat coronavirus HKU9-2 | DBatVir/Genebank |
| 15 | EF065515 | Bat coronavirus HKU9-3 | DBatVir/Genebank |
| 16 | EF065516 | Bat coronavirus HKU9-4 | DBatVir/Genebank |
| 17 | HM211098 | Bat coronavirus HKU9-5-1 | DBatVir/Genebank |
| 18 | HM211099 | Bat coronavirus HKU9-5-2 | DBatVir/Genebank |
| 19 | JX993987 | Bat coronavirus Rp/Shaanxi2011 | DBatVir/Genebank |
| 20 | DQ648856 | Bat coronavirus (BtCoV/273/2005) | DBatVir/Genebank |
| 21 | DQ648857 | Bat coronavirus (BtCoV/279/2005) | DBatVir/Genebank |
| 22 | KF636752 | Bat Hp-betacoronavirus/Zhejiang2013 | DBatVir/Genebank |
| 23 | DQ022305 | Bat SARS coronavirus HKU3-1 | DBatVir/Genebank |
| 24 | GQ153545 | Bat SARS coronavirus HKU3-10 | DBatVir/Genebank |
| 25 | GQ153546 | Bat SARS coronavirus HKU3-11 | DBatVir/Genebank |
| 26 | GQ153547 | Bat SARS coronavirus HKU3-12 | DBatVir/Genebank |
| 27 | GQ153548 | Bat SARS coronavirus HKU3-13 | DBatVir/Genebank |
| 28 | DQ084199 | Bat SARS coronavirus HKU3-2 | DBatVir/Genebank |
| 29 | DQ084200 | Bat SARS coronavirus HKU3-3 | DBatVir/Genebank |
| 30 | GQ153539 | Bat SARS coronavirus HKU3-4 | DBatVir/Genebank |
| 31 | GQ153540 | Bat SARS coronavirus HKU3-5 | DBatVir/Genebank |
| 32 | GQ153541 | Bat SARS coronavirus HKU3-6 | DBatVir/Genebank |
| 33 | GQ153542 | Bat SARS coronavirus HKU3-7 | DBatVir/Genebank |
| 34 | GQ153543 | Bat SARS coronavirus HKU3-8 | DBatVir/Genebank |
| 35 | GQ153544 | Bat SARS coronavirus HKU3-9 | DBatVir/Genebank |
| 36 | DQ412042 | Bat SARS coronavirus Rf1 | DBatVir/Genebank |
| 37 | DQ412043 | Bat SARS coronavirus Rm1 | DBatVir/Genebank |
| 38 | DQ071615 | Bat SARS coronavirus Rp3 | DBatVir/Genebank |
| 39 | KC881006 | Bat SARS-like coronavirus Rs3367 | DBatVir/Genebank |
| 40 | KC881005 | Bat SARS-like coronavirus RsSHC014 | DBatVir/Genebank |
| 41 | KF367457 | Bat SARS-like coronavirus WIV1 | DBatVir/Genebank |
| 42 | KP886808 | Bat SARS-like coronavirus YNLF_31C | DBatVir/Genebank |
| 43 | KP886809 | Bat SARS-like coronavirus YNLF_34C | DBatVir/Genebank |
| 44 | KJ473821 | BtVs-BetaCoV/SC2013 | DBatVir/Genebank |
| 45 | MK211374 | Coronavirus BtRl-BetaCoV/SC2018 | DBatVir/Genebank |
| 46 | MK211375 | Coronavirus BtRs-BetaCoV/YN2018A | DBatVir/Genebank |
| 47 | MK211376 | Coronavirus BtRs-BetaCoV/YN2018B | DBatVir/Genebank |
| 48 | MK211377 | Coronavirus BtRs-BetaCoV/YN2018C | DBatVir/Genebank |
| 49 | MK211378 | Coronavirus BtRs-BetaCoV/YN2018D | DBatVir/Genebank |
| 50 | MK211379 | Coronavirus BtRt-BetaCoV/GX2018 | DBatVir/Genebank |
| 51 | KC869678 | Coronavirus Neoromicia/PML-PHE1/RSA/2011 | DBatVir/Genebank |
| 52 | MG596802 | MERS-related Bat-CoV/H.savii/Italy/206645-40/2011 | DBatVir/Genebank |
| 53 | MG596803 | MERS-related Bat-CoV/P.khulii/Italy/206645-63/2011 | DBatVir/Genebank |
| 54 | MF593268 | MERS-related Neoromicia/5038 | DBatVir/Genebank |
| 55 | MG021451 | MERS-related NL13845 | DBatVir/Genebank |
| 56 | MG987420 | MERS-related NL13892 | DBatVir/Genebank |
| 57 | MG021452 | MERS-related NL140422 | DBatVir/Genebank |
| 58 | MG987421 | MERS-related NL140455 | DBatVir/Genebank |
| 59 | MN611520 | Pipistrellus abramus bat coronavirus HKU5-related isolate BY140568 | DBatVir/Genebank |
| 60 | MG762674 | Rousettus bat coronavirus HKU9 isolate Rousettus spp/Jinghong/2009 | DBatVir/Genebank |
| 61 | FJ588686 | Bat SARS CoV Rs672/2006 | DBatVir/Genebank |
| 62 | KT444582 | SARS-like coronavirus WIV16 | DBatVir/Genebank |
| 63 | KY417142 | Bat SARS-like coronavirus isolate As6526 | DBatVir/Genebank |
| 64 | MG772933 | Bat SARS-like coronavirus isolate bat-SL-CoVZC45 | DBatVir/Genebank |
| 65 | MG772934 | Bat SARS-like coronavirus isolate bat-SL-CoVZXC21 | DBatVir/Genebank |
| 66 | KY417145 | Bat SARS-like coronavirus isolate Rf4092 | DBatVir/Genebank |
| 67 | KY417143 | Bat SARS-like coronavirus isolate Rs4081 | DBatVir/Genebank |
| 68 | KY417144 | Bat SARS-like coronavirus isolate Rs4084 | DBatVir/Genebank |
| 69 | KY417146 | Bat SARS-like coronavirus isolate Rs4231 | DBatVir/Genebank |
| 70 | KY417147 | Bat SARS-like coronavirus isolate Rs4237 | DBatVir/Genebank |
| 71 | KY417148 | Bat SARS-like coronavirus isolate Rs4247 | DBatVir/Genebank |
| 72 | KY417149 | Bat SARS-like coronavirus isolate Rs4255 | DBatVir/Genebank |
| 73 | KY417150 | Bat SARS-like coronavirus isolate Rs4874 | DBatVir/Genebank |
| 74 | KY417151 | Bat SARS-like coronavirus isolate Rs7327 | DBatVir/Genebank |
| 75 | KY417152 | Bat SARS-like coronavirus isolate Rs9401 | DBatVir/Genebank |
| 76 | KY352407 | SARS-related coronavirus strain BtKY72 | DBatVir/Genebank |
| 77 | MN611519 | Tylonycteris pachypus bat coronavirus HKU4-related isolate GZ131656 | DBatVir/Genebank |
| 78 | MN996532.1 | Bat coronavirus RaTG13 | GenBank |
| 79 | EPI_ISL_410544 | BetaCoV/pangolin/Guangdong/P2S/2019 | GISAID |
| 80 | EPI_ISL_410539 | BetaCoV/pangolin/Guangxi/P1E/2017 | GISAID |
| 81 | EPI_ISL_410538 | BetaCoV/pangolin/Guangxi/P4L/2017 | GISAID |
| 82 | EPI_ISL_410543 | BetaCoV/pangolin/Guangxi/P3B/2017 | GISAID |
| 83 | EPI_ISL_410542 | BetaCoV/pangolin/Guangxi/P2V/2017 | GISAID |
| 84 | EPI_ISL_410541 | BetaCoV/pangolin/Guangxi/P5E/2017 | GISAID |
| 85 | EPI_ISL_410540 | BetaCoV/pangolin/Guangxi/P5L/2017 | GISAID |
| 86 | EPI_ISL_412860 | BetaCoV/pangolin/China/MP789/2019 | GISAID |
| 87 | EPI_ISL_410721 | BetaCoV/pangolin/Guangdong/1/2019 | GISAID |
| 88 | NC_045512.2 | Severe acute respiratory syndrome coronavirus 2 isolate Wuhan-Hu-1 | GenBank |

Supplementary Table 2. Accession numbers of the ORF1ab of the 24 viruses used in this study.

| **No.** | **Accession number** | **Virus** |
| --- | --- | --- |
| 1 | YP_009724389.1 | SARS-CoV-2 |
| 2 | NP_828849.2 | SARS |
| 3 | QIA48613.1 | Pangolin coronavirus (2017) |
| 4 | QIG55944.1 | Pangolin coronavirus (2019) |
| 5 | QHR63299.1 | Bat coronavirus RaTG13 |
| 6 | AVP78030.1 | Bat-SL-CoVZC45 |
| 7 | AVP78041.1 | Bat SARS-like coronavirus |
| 8 | AGC74171.1 | Bat coronavirus Cp/Yunnan2011 |
| 9 | QDF43829.1 | Coronavirus BtRs-BetaCoV/YN2018C |
| 10 | NP_073549.1 | Human coronavirus 229E |
| 11 | YP_003766.2 | Human coronavirus NL63 |
| 12 | YP_009555238.1 | Human coronavirus OC43 |
| 13 | YP_173236.1 | Human coronavirus HKU1 |
| 14 | YP_009047202.1 | MERS |
| 15 | AFH35028.1 | Infectious bronchitis virus Sczy3 |
| 16 | AJT47868.1 | Infectious bronchitis virus ck/CH/LHB/121041 |
| 17 | YP_001876435.1 | Beluga whale coronavirus SW1 |
| 18 | YP_009755895.1 | Canada goose coronavirus |
| 19 | YP_005352853.1 | Magpie-robin coronavirus HKU18 |
| 20 | YP_002308505.1 | Munia coronavirus HKU13-3514 |
| 21 | YP_005352870.1 | Wigeon coronavirus HKU20 |
| 22 | YP_009513020.1 | Porcine coronavirus HKU15 |
| 23 | YP_001718610.1 | Miniopterus bat coronavirus HKU8 |
| 24 | YP_006908641.2 | Rousettus bat coronavirus HKU10 |

Supplementary Table 3. Key software or databases used in this study.

| **No.** | **Software** | **Source** |
| --- | --- | --- |
| 1 | EMBOSS Needle | https://www.ebi.ac.uk/Tools/psa/emboss_needle/ |
| 2 | Clustal Omega | http://www.clustal.org/omega/ |
| 3 | UGENE | http://ugene.net/ |
| 4 | MEGA X | https://megasoftware.net/ |
| 5 | GERP++ | http://mendel.stanford.edu/SidowLab/downloads/gerp/ |
| 6 | G4CatchAll | http://homes.ieu.edu.tr/odoluca/G4Catchall/ |
| 7 | pqsfinder | http://www.bioconductor.org/packages/release/bioc/html/pqsfinder.html |
| 8 | QGRS Mapper | https://bioinformatics.ramapo.edu/QGRS/index.php |
| 9 | BEDTools | https://bedtools.readthedocs.io/en/latest/ |
| 10 | cG/cC scoring* | https://github.com/rongxinzh/cGcC |
| 11 | Swiss Model | https://swissmodel.expasy.org/ |
| 12 | PyMOL | https://pymol.org/2/ |
| 13 | RNAStructuromeDB | https://structurome.bb.iastate.edu/ |
| 14 | GISAID | https://www.gisaid.org/ |
| 15 | DBatVir | <http://www.mgc.ac.cn/DBatVir/> |

* Customized script

Supplementary Table 4. cG/cC score of potential RNA G-quadruplexes with flanking sequences in SARS-CoV-2.

| **No.** | **Sequence** | **cG/cC score** |
| --- | --- | --- |
| 1 | AAGAGATCGAAAGTTGGTTGGTTTGTTACCTGGGAAGGTATAAACCTTTAAT | 3 |
| 2 | GACGTGCTCGTACGTGGCTTTGGAGACTCCGTGGAGGAGGTCTTATCAGAGGCAC | 2.29 |
| 3 | CTCGTACGTGGCTTTGGAGACTCCGTGGAGGAGGTCTTATCAGAGGCAC | 2.23 |
| 4 | CTTCTTCGTAAGAACGGTAATAAAGGAGCTGGTGGCCATAGTTACGGCGC | 2.08 |
| 5 | AAGATAATTTCTTTTGGGGCTTTTAGAGGCATGAGTAGGCCAGTTTCTTCTCTG | 3.4 |
| 6 | GTTTACCTTAAACATGGAGGAGGTGTTGCAGGAGCCTTAAATAAGGC | 2.18 |
| 7 | CACTTACCCGGGTCAGGGTTTAAATGGTTACACTGTAGAGGAGGCAAAGACAGTGCTTA | 2.06 |
| 8 | ACTTACCCGGGTCAGGGTTTAAATGGTTACACTGTAGAGGAGGCAAAGACAGTGCTTA | 2.18 |
| 9 | TAACTTCACCATCTAGGTGGAATGTGGTAGGATTACTAGTGTAATA | 2.38 |
| 10 | TCGAAGCTTGCGTTTGGATATGGTTGGTTTGGTACAAGATCAATTGG | 5 |
| 11 | TCAAGTGAAATCATAGGATACAAGGCTATTGATGGTGGTGTCACTCGTGACAT | 2.75 |
| 12 | AGCTGAGGTGATAGAGGTTTGTGGTGGTTGGTAAAGAACATCAGAA | 9 |
| 13 | CTGCAAAACAGCTGAGGTGATAGAGGTTTGTGGTGGTTGGTAAAGAACATCAGAA | 4.67 |
| 14 | CTGCAAAACAGCTGAGGTGATAGAGGTTTGTGGTGGTTGGTAAAGAACATC | 4.5 |
| 15 | TAATCATAATTTCTTGGTACAGGCTGGTAATGTTCAACTCAGGGTTATTGGACATTCT | 3 |
| 16 | TAATTTCTTGGTACAGGCTGGTAATGTTCAACTCAGGGTTATTGGACATTCTATGCAAAA | 3.11 |
| 17 | GTCTGTACCGTCTGCGGTATGTGGAAAGGTTATGGCTGTAGTTGTGATCA | 2.78 |
| 18 | CCTAGGATTCTTGATGGATCTGGGTAAGGAAGGTACACATAATCATCA | 2.7 |
| 19 | GACATGTACGTGCATGGATTGGCTTCGATGTCGAGGGGTGTCATGCTACTAGA | 4.22 |
| 20 | ATTTGCTATGCAAATGGCTTATAGGTTTAATGGTATTGGAGTTACACAGAATGT | 4.2 |
| 21 | AGCAGTATATAAAATGGCCATGGTACATTTGGCTAGGTTTTATAGCTGGCTT | 2.56 |
| 22 | CATGACTACCAGATTGGTGGTTATACTGAAAAATGGGAATCTGGAGTAAAAGACTGTGT | 3.22 |
| 23 | TTTACAGAATAAATTGGATCACCGGTGGAATTGCTATCGCAATGGCTTGTCTTGTAGGCT | 2.08 |
| 24 | TCACTTTCTAGAAGCGGTCTGGTCAGAATAGTGCCATGGAGTGGCACGTTGAGAAGAAT | 2.17 |

Supplementary Table 5. Experimental confirmed viral two-quartet G-quadruplexes *in vitro*.

| **No.** | **Sequence** | **Virus** | **Source** | **Validation method** |
| --- | --- | --- | --- | --- |
| 1 | GGAGAACGGGATGGTTAAGG | Nipah virus | Prativa Majee et al. | NMR spectroscopy, EMSA, CD spectroscopy, DMS footprinting assays |
| 2 | GGTAAACGGTGTTTGGATTTGGTGGGG | Nipah virus | Prativa Majee et al. | NMR spectroscopy, EMSA, CD spectroscopy, DMS footprinting assays |
| 3 | GGAAGAGTGATAGGACTCTATGGCAATGGGG | Zika virus | Aaron M. Fleming et al. | Native-gel analysis, NMR spectroscopy, CD spectroscopy |
| 4 | GGAGGTGGGACGGG | Zika virus | Aaron M. Fleming et al. | Native-gel analysis, NMR spectroscopy, CD spectroscopy |
| 5 | GGATGTGGCAGAGGGGGCTGG | Zika virus | Aaron M. Fleming et al. | Native-gel analysis, NMR spectroscopy, CD spectroscopy |
| 6 | GGCGGCCGGTGTGGGG | Zika virus | Aaron M. Fleming et al. | Native-gel analysis, NMR spectroscopy, CD spectroscopy |
| 7 | GGGGCAGGAGCAGGAGG | Epstein-Barr virus | Pierre Murat et al. | NMR spectroscopy, CD spectroscopy, UV thermal difference spectroscopy |
| 8 | GGCGGCGGCGG | Pseudorabies virus | Yashu Zhang et al. | CD spectroscopy, TDS spectroscopy, NMR spectroscopy |
| 9 | GGCGGCGGAGG | Pseudorabies virus | Yashu Zhang et al. | CD spectroscopy, TDS spectroscopy, NMR spectroscopy |
| 10 | GGCGGAGGTGG | Pseudorabies virus | Yashu Zhang et al. | CD spectroscopy, TDS spectroscopy, NMR spectroscopy |
| 11 | GGAGGTGGCGG | Pseudorabies virus | Yashu Zhang et al. | CD spectroscopy, TDS spectroscopy, NMR spectroscopy |
| 12 | GGTGGCGGCGG | Pseudorabies virus | Yashu Zhang et al. | CD spectroscopy, TDS spectroscopy, NMR spectroscopy |
| 13 | GGCTCGGCGGCGG | Pseudorabies virus | Yashu Zhang et al. | CD spectroscopy, TDS spectroscopy, NMR spectroscopy |
| 14 | GGACGAGGAGGAGGACGAGGAGGACGAGGAGG | Manatee papillomaviruses | Maryam Zahin et al. | Analytical ultracentrifugation, CD spectroscopy, Thermal denaturation, Thioflavin binding |
| 15 | GGAGCAGGAGAAGGAGGAGGAGGAGGACGAGGAGGACGAGGAGGAGGACGAGGAGGACGAGGAGG | Manatee papillomaviruses | Maryam Zahin et al. | Analytical ultracentrifugation, CD spectroscopy, Thermal denaturation, Thioflavin binding |
| 16 | GGAGGAGGAGG | Manatee papillomaviruses | Maryam Zahin et al. | Analytical ultracentrifugation, CD spectroscopy, Thermal denaturation, Thioflavin binding |

Supplementary Table 6. cG/cC score of the experimental supported G-quadruplexes with flanking sequences in other viruses.

| **No.** | **Sequence** | **Virus** | **cG/cC score** |
| --- | --- | --- | --- |
| 1 | CGGTAAATATTTTAAGGAGAACGGGATGGTTAAGGATGAGCACGAACTTT | Nipha virus | 6.0 |
| 2 | ATACAGCACTACAGTGGTAAACGGTGTTTGGATTTGGTGGGGTCCAGACATTGGTCA | Nipha virus | 4.0 |
| 3 | ATCCTAGACAAATGTGGAAGAGTGATAGGACTCTATGGCAATGGGGTTGTGATCAAGAATG | Zika virus | 4.56 |
| 4 | CTGGTTAAGAGACGTGGAGGTGGGACGGGAGAGACTCTGGGAGA | Zika virus | 9.6 |
| 5 | AAGGTTGTTGACCTCGGATGTGGCAGAGGGGGCTGGAGCTATTATGCCGCC | Zika virus | 3.63 |
| 6 | AGATCGCCGAACTTCGGCGGCCGGTGTGGGGAAATCCATGGTTTCT | Zika virus | 2.35 |
| 7 | GCCGGAGGCGGCTGCGGCGGCGGCGGAGGTGGCGGCGGAGG | Pseudorabies virus | 4.17 |
| 8 | GGCGGAGGTGGAAGCGGCGGCGGCGGCCGCGGCGGAGGGCT | Pseudorabies virus | 4.33 |
| 9 | GGAGGCGGCTGCGGCGGCGGCGGAGGTGGCGGCGGAGGTGG | Pseudorabies virus | 6.63 |
| 10 | GGCGGCGGCGGAGGTGGCGGCGGAGGTGGCGGCGGAGGTGG | Pseudorabies virus | 8.0 |
| 11 | GGTGGCGGCGGAGGTGGCGGCGGAGGTGGCGGCGGAGGTGG | Pseudorabies virus | 9.33 |
| 12 | GGTGGCGGCGGAGGTGGCGGCGGAGGTGGAAGCGGCGGCGG | Pseudorabies virus | 7.57 |
| 13 | CGCGGCGGAGGGCTCGGCGGCGGAGGATCGTCCCGGTCCCC | Pseudorabies virus | 1.05 |
| 14 | GGCGGCTGCGGCGGCGGCGGAGGTGGCGGCGGAGGTGGCGG | Pseudorabies virus | 5.89 |
| 15 | GGCGGCGGAGGTGGCGGCGGAGGTGGCGGCGGAGGTGGCGG | Pseudorabies virus | 8.0 |
| 16 | GGCGGCGGAGGTGGCGGCGGAGGTGGCGGCGGAGGTGGAAG | Pseudorabies virus | 8.83 |
| 17 | GGCGGCGGAGGTGGCGGCGGAGGTGGAAGCGGCGGCGGCGG | Pseudorabies virus | 6.625 |
| 18 | GGCTGCGGCGGCGGCGGAGGTGGCGGCGGAGGTGGCGGCGG | Pseudorabies virus | 5.89 |
| 19 | GGCGGAGGTGGCGGCGGAGGTGGCGGCGGAGGTGGCGGCGG | Pseudorabies virus | 8.0 |
| 20 | GGCGGAGGTGGCGGCGGAGGTGGCGGCGGAGGTGGAAGCGG | Pseudorabies virus | 8.83 |
| 21 | TGCGGCGGCGGCGGAGGTGGCGGCGGAGGTGGCGGCGGAGG | Pseudorabies virus | 6.63 |
| 22 | GGAGGTGGCGGCGGAGGTGGCGGCGGAGGTGGCGGCGGAGG | Pseudorabies virus | 9.33 |
| 23 | GGAGGTGGCGGCGGAGGTGGCGGCGGAGGTGGAAGCGGCGG | Pseudorabies virus | 8.83 |
| 24 | GCGGCCGCGGCGGAGGGCTCGGCGGCGGAGGATCGTCCCGGTC | Pseudorabies virus | 1.96 |
| 25 | GGAGGAGGACGAGGAGGACGAGGAGGAGGACGAGGAGGACGAGGAGGGAGCTGTACCACCAC | Manatee Papilloma virus | 4.29 |
| 26 | GCCCAAGGGCACCGAGGAGCAGGAGAAGGAGGAGGAGGAGGACGAGGAGGACGAGGAGGAGGACGAGGAGGACGAGGAGGGAGCTGTACCACCAC | Manatee Papilloma virus | 3.0 |
| 27 | CGAGGAGCAGGAGAAGGAGGAGGAGGAGGACGAGGAGGACG | Manatee Papilloma virus | 10.25 |
| 28 | GGGGCAGGAGCAGGAGGGGCAGGAGCAGGAGGAGGGGCAGGAGCAGG | Epstein-Barr virus | 15.17 |
| 29 | GCAGGAGCAGGAGGAGGGGCAGGAGCAGGAGGAGGGGCAGGAGGGGC | Epstein-Barr virus | 15.17 |
| 30 | GCAGGAGGGGCAGGAGGGGCAGGAGCAGGAGGAGGGGCAGGAGCAGG | Epstein-Barr virus | 15.17 |
| 31 | GGAGGAGGGGCAGGAGGGGCAGGAGCAGGAGGGGGGCAGGAGGGGCA | Epstein-Barr virus | 28.2 |
| 32 | GCAGGAGGGGCAGGAGGGGCAGGAGCAGGAGGAGGGGCAGGAGGGGC | Epstein-Barr virus | 17.67 |
| 33 | GGAGGAGGGGCAGGAGGGGCAGGAGCAGGAGGAGGGGCAGGAGGGGC | Epstein-Barr virus | 21.8 |

Since some G-quadruplexes may appear multiple times in a specific viral genome, the number of G-quadruplex with its flanking sequences given in the table is more than the number of G-quadruplexes listed in the Supplementary Table 5.
